# How to overcome the stagnation of the first dose vaccine coverage curve against coronavirus disease 2019 in Brazil?

**DOI:** 10.1590/0037-8682-0722-2021

**Published:** 2022-06-06

**Authors:** Raphael Mendonça Guimarães, Diego Ricardo Xavier, Raphael de Freitas Saldanha, Mônica de Avelar Figueiredo Mafra Magalhães

**Affiliations:** 1 Fundação Oswaldo Cruz, Escola Nacional de Saúde Pública, Rio de Janeiro, RJ, Brasil.; 2 Fundação Oswaldo Cruz, Instituto de Comunicação e Informação em Ciência e Tecnologia, Rio de Janeiro, RJ, Brasil.

**Keywords:** Vaccination, COVID-19, Spatial analysis, Health management, Coronavirus infections, Time series analysis

## Abstract

**Background:**

A large percentage of the population has not yet started vaccination, for which the increase in coverage is almost null.

**Methods:**

We used segmented regression analysis to estimate trends in the first dose coverage curve.

**Results:**

There has been a slowdown in the application of the first doses in Brazil since epidemiological week 36 (average percent change [APC] 0.83%, 95% confidence interval [CI] 0.75-0.91%), with a trend close to stagnation.

**Conclusions:**

It is important to develop strategies to increase access to vaccination posts. Furthermore, it is recommended to expand vaccination to children, thereby increasing the eligible population.

Historically, vaccines have been among the most effective and efficient technical tools for controlling infectious diseases^1^. However, the success of this strategy is the result of technological advances in producing immunizing agents and logistical support strategies for public health, including distribution, application, and population mobilization, to resolve uncertainties regarding the risks and benefits of the vaccine. As coronavirus disease 2019 (COVID-19) vaccination is rolled out globally, scientists and decision-makers need to investigate the scale and determinants of expanding vaccine coverage in each location^2^.

Vaccination in Brazil against COVID-19 started on January 17, 2021^3^. Although emerging challenges, such as the continental dimension of the country, prioritizing the most vulnerable groups, and irregular distribution of doses, complete vaccination coverage continues to grow. Currently, the percentage of the population with full vaccination coverage is 74%. However, since September 2021, the pace of first-dose vaccination in Brazil has slowed down. Between October 9 (when the country reached 70% of those vaccinated with the first dose) and the subsequent 60 days, it showed constant growth and was close to zero, ranging between 70.04 and 74.95%; therefore, with an increase of 0.08% per day^4^. This observation requires special attention, as stagnation of the growth curve for the first dose suggests population saturation for vaccination. This article describes the temporal evolution of first dose vaccination coverage against COVID-19 in Brazil and its states.

According to the epidemiological week (EW), we performed the analysis with vaccination coverage data by the federation unit. The reference date was the last day of each EW. We considered the number of doses applied in the numerator to estimate vaccination coverage considering the position (first or second dose). We initially used the total population as the denominator, a standardized indicator for monitoring vaccination coverage. Population estimates were obtained from the projections of the Brazilian Bureau of Statistics (IBGE).

The focus of the analysis was the coverage of the first dose, which showed signs of stagnation in all federation units. Therefore, we performed a coverage trend analysis using segmented regression modeling. (Joinpoint regression). The statistical model assumes a trend between the inflection points (union points). Whenever there is a significant change between a point join and the next point over time, we assume that it is an inflection point, and from that point on, there is a new regression trend. This method uses an approximate Monte Carlo permutation to calculate the *P*-value under the null hypothesis each time. Using Bonferroni correction for the global alpha level, we maintained the overall asymptotic significance level to determine the location of the junction points on the time scale and calculated the weekly percent change (average percent change [APC]) for each defined period.

We used Poisson distribution parameters with robust variance to guarantee the homoscedasticity assumption. The selection of the number of inflection points was performed automatically using Joinpoint Regression Program 4.8.0.1. A confidence level of 95 % (95% CI) was considered. 

Based on this level of vaccination coverage, we analyzed the spatial distribution of the first and second dose coverage among the states. We then discount the population under 12 years of age to estimate the current coverage by considering only the eligible population. The reference period for the analysis was epidemiological week 47, which corresponds to the last week of November 2021.

Brazil has four well-marked phases in the temporal evolution of first-dose vaccination coverage. The first phase (EW 3-10), with expected slow progression, resulted from the accommodation of the start of vaccination and the lack of immunization in the period. The second phase starts after approximately 10 weeks when it reaches the target older adult population below 70 years of age (EW 10-22). The third phase (EW 22-36) accelerated the speed of coverage, which occurs during the initiation of vaccination for adults under 60 years of age. Finally, deceleration occurred at the beginning of September (EW 36-47). All the states also presented four phases. However, the inflection points that gave rise to each of them varied so that these phases were quite different.

The segmented regression model allowed the estimation of the increment speed for each period (Table 1). For the four phases mentioned, the weekly percentage variations were 0.53% (95% CI 0.37%-0.59%), 1.59% (95% CI 1.51-1.67), 3.07 (95% CI 3.01-3.13), and 0.83 (95% CI 0.75-0.91). Most states follow this trend, varying only the rate of increase in coverage, which was systematically higher in the Southern and Southeastern regions. We highlight São Paulo and the Federal District at different speeds from the other states. Furthermore, we emphasize that the Northern states present a percentage variation of the first phase greater than the current moment, which suggests a faster deceleration in these places. In units of the federation, where the coverage of the first dose was higher, the difference in the second dose coverage was smaller, suggesting that the population loss between doses was small.

**TABLE 1:** Vaccination coverage indicators by unit of the federation in Brazil, 2021.

FU^a^	Vaccine coverage		Target^b^	D1 - D2^c^	Joinpoint (1^st^ dose)			
	1^st^ dose	2^nd^ dose			Period (EW^d^)	APC^e^ (%)	95% CI^f^	***P*-value**
Acre	61.82	45.66	79.67	16.16	3 to 22	0.83	0.77-0.89	<0.001
					22 to 27	3.89	3.26-4.52	<0.001
					27 to 35	2.35	2.17-2.53	<0.001
					37 to 47	0.49	0.33-0.65	<0.001
Alagoas	69.26	52.08	83.23	17.18	3 to 10	0.39	0.08-0.70	0.024
					10 to 23	1.48	1.34-1.62	<0.001
					23 to 39	2.50	2.40-2.60	<0.001
					39 to 47	0.87	0.62-1.12	<0.001
Amazonas	62.82	48.49	79.13	14.33	3 to 22	0.93	0.89-0.97	<0.001
					22 to 27	4.07	3.58-4.56	<0.001
					27 to 37	1.93	1.79-2.07	<0.001
					37 to 47	0.46	0.34-0.58	<0.001
Bahia	71.36	54.14	84.95	17.22	3 to 9	0.44	0.07-0.81	0.029
					9 to 25	1.65	1.55-1.75	<0.001
					25 to 38	2.72	2.58-2.86	<0.001
					38 to 47	0.75	0.55-0.95	<0.001
Amapá	57.37	36.72	79.85	20.65	3 to 11	0.46	0.30-0.62	<0.001
					11 to 24	1.04	0.96-1.12	<0.001
					24 to 36	2.71	2.61-2.81	<0.001
					36 to 47	0.71	0.61-0.81	<0.001
Ceará	73.39	62.57	84.54	10.82	3 to 9	0.57	-0.04-1.18	0.081
					9 to 21	1.23	0.99-1.47	<0.001
					21 to 38	2.78	2.64-2.92	<0.001
					38 to 47	0.85	0.52-1.18	<0.001
Distrito Federal	73.76	63.38	85.94	10.38	3 to 9	1.02	0.90-1.14	<0.001
					9 to 30	2.32	2.10-2.54	<0.001
					30 to 33	7.72	4.88-10.56	<0.001
					33 to 47	0.76	0.62-0.90	<0.001
Espírito Santo	74.73	62.75	84.67	11.98	3 to 10	0.44	0.05-0.83	0.034
					10 to 20	1.85	1.60-2.10	<0.001
					20 to 38	2.65	2.55-2.75	<0.001
					38 to 47	0.60	0.35-0.85	<0.001
Goiás	71.97	55.22	84.14	16.75	3 to 12	0.53	0.37-0.69	<0.001
					12 to 22	1.59	1.43-1.75	<0.001
					22 to 38	2.81	2.73-2.89	<0.001
					38 to 47	0.71	0.55-0.87	<0.001
Maranhão	61.62	46.61	81.99	15.01	3 to 21	0.98	0.88-1.08	<0.001
					21 to 25	3.94	2.51-5.37	<0.001
					25 to 37	1.97	1.79-2.15	<0.001
					37 to 47	0.71	0.49-0.93	<0.001
Mato Grosso	70.75	54.76	82.92	15.99	3 to 11	0.37	0.21-0.53	<0.001
					11 to 23	1.34	1.24-1.44	<0.001
					23 to 36	3.06	2.98-3.14	<0.001
					36 to 47	1.13	1.03-1.23	<0.001
Mato Grosso do Sul	71.24	69.81	83.12	1.43	3 to 11	0.62	0.35-0.89	<0.001
					11 to 31	2.34	2.26-2.42	<0.001
					31 to 34	4.08	1.51-6.65	0.003
					34 to 47	0.43	0.29-0.57	<0.001
Minas Gerais	76.46	63.89	86.39	12.57	3 to 11	0.53	0.37-0.69	<0.001
					11 to 24	1.79	1.71-1.87	<0.001
					24 to 37	3.09	3.01-3.17	<0.001
					37 to 47	0.95	0.83-1.07	<0.001
Pará	60.19	39.89	81.89	20.3	3 to 22	0.98	0.88-1.08	<0.001
					22 to 25	3.68	0.01-7.35	0.057
					25 to 42	1.73	1.59-1.87	<0.001
					42 to 47	0.57	-0.25-1.39	0.186
Paraíba	75.45	58.47	84.86	16.98	3 to 9	0.58	0.19-0.97	0.007
					9 to 25	1.52	1.42-1.62	<0.001
					25 to 36	3.34	3.16-3.52	<0.001
					36 to 47	1.05	0.89-1.21	<0.001
Paraná	77.08	65.79	85.22	11.29	3 to 10	0.46	0.22-0.70	<0.001
					10 to 21	1.61	1.47-1.75	<0.001
					21 to 35	3.18	3.08-3.28	<0.001
					35 to 47	0.97	0.87-1.07	<0.001
Pernambuco	74.00	58.44	84.23	15.56	3 to 9	0.50	0.25-0.75	<0.001
					9 to 21	1.28	1.18-1.38	<0.001
					21 to 37	2.82	2.76-2.88	<0.001
					37 to 47	1.08	0.96-1.20	<0.001
Piauí	74.87	58.40	84.19	16.47	3 to 10	0.46	0.17-0.75	<0.001
					10 to 22	1.36	1.20-1.52	<0.001
					22 to 37	2.75	2.65-2.85	<0.001
					37 to 47	1.50	1.32-1.68	<0.001
Rio de Janeiro	74.15	60.15	86.00	14.00	3 to 11	0.54	0.36-0.72	<0.001
					11 to 20	1.41	1.23-1.59	<0.001
					20 to 38	2.84	2.78-2.90	<0.001
					38 to 47	0.71	0.57-0.85	<0.001
Rio Grande do Norte	72.34	59.97	85.13	12.37	3 to 10	0.46	0.19-0.73	0.003
					10 to 22	1.47	1.31-1.59	<0.001
					22 to 37	2.96	2.86-3.06	<0.001
					37 to 47	0.73	0.57-0.89	<0.001
Rio Grande do Sul	72.27	67.94	86.56	9.33	3 to 11	0.62	0.37-0.87	<0.001
					11 to 24	2.05	1.93-2.17	<0.001
					24 to 33	3.06	2.94-3.18	<0.001
					33 to 47	0.92	0.82-1.02	0.001
Rondônia	66.43	52.90	83.25	13.53	3 to 11	0.47	0.29-0.65	0.002
					11 to 22	1.01	0.87-1.15	<0.001
					22 to 36	3.23	3.15-3.31	<0.001
					36 to 47	0.59	0.47-0.71	<0.001
Roraima	55.61	38.45	80.35	17.16	3 to 22	0.70	0.64-0.76	<0.001
					22 to 30	2.74	2.47-3.01	<0.001
					30 to 38	1.78	1.51-2.05	<0.001
					38 to 47	0.70	0.52-0.88	<0.001
São Paulo	81.41	74.80	85.69	6.61	3 to 13	0.96	0.82-1.10	<0.001
					13 to 22	1.86	1.66-2.06	<0.001
					22 to 35	3.88	3.78-3.98	<0.001
					35 to 47	0.47	0.37-0.57	<0.001
Santa Catarina	78.14	67.39	85.68	10.75	3 to 10	0.47	0.20-0.74	0.001
					10 to 21	1.65	1.49-1.81	<0.001
					21 to 36	3.11	3.01-3.21	<0.001
					36 to 47	0.99	0.85-1.13	<0.001
Sergipe	73.04	60.40	83.96	12.64	3 to 9	0.37	-0.04-0.78	0.09
					9 to 21	1.40	-0.17-2.97	<0.001
					21 to 38	2.89	2.79-2.99	<0.001
					38 to 47	0.54	0.32-0.76	<0.001
Tocantins	65.07	49.47	82.80	15.6	3 to 11	0.48	0.34-0.62	<0.001
					11 to 22	1.23	1.13-1.33	<0.001
					22 to 38	2.70	2.66-2.74	<0.001
					38 to 47	0.54	0.42-0.66	<0.001
**Brazil**	**74.42**	**62.20**	**84.86**	**12.22**	**3 to 10**	**0.53**	**0.37-0.69**	**<0.001**
					**10 to 22**	**1.59**	**1.51-1.67**	**<0.001**
					**22 to 36**	**3.07**	**3.01-3.13**	**<0.001**
					**36 to 4**7	**0.83**	**0.75-0.91**	**<0.001**

**Legend:** ^a^ FU: Federal Unit; ^b^ Population Target: eligible population (above 11 years old at time of analysis, based on IBGE projection); ^c^ D1 - D2: difference in first and second dose coverage; ^d^ EW: Epidemiological Week; ^e^ APC: Average Percent Change; ^f^ 95% CI: 95% Confidence Interval. **Source:** MonitoraCovid. 2021.

Regarding the population, 84.86% of people in Brazil are eligible to apply for the first dose of the vaccine, that is, over 11 years of age. In late November, the first dose coverage was 74.42%, and the full coverage (two doses or one single dose) was 62.2%. In units of the federation where the first dose coverage was higher, the difference in the second dose coverage was smaller, suggesting that the population loss between doses was slight. In addition, places with a small difference between the first dose coverage and the complete vaccination schedule had the largest adult population. This analysis suggests two distinct scenarios. On the one hand, places with older people have higher full vaccination coverage and are already close to the saturation of the eligible population. In contrast, places with younger populations have lower complete vaccination coverage. This indicates that the progression of vaccination coverage is strongly dependent on the inclusion of younger age groups. 

The states in the Northern region had a more rejuvenated population, which may partially explain the lower coverage in these states. To correct this difference, we proceeded to the second stage of the analysis, deflating the denominator of the coverage rate and including only the population eligible for vaccination up to the reference period used (12 years or more). Again, we observe the persistence of regional inequality. The states in the North and Northeast regions had the worst coverage, both in the first (Figure 1a ) and second doses (Figure 1b). Taking the country's coverage as a cut-off point for comparison, these states remain in the quadrant marked by lower coverage (Figure 1c), making it evident that national values are inflated along the Center-south axis. São Paulo and Amapá had the highest and lowest vaccination coverage, respectively, in the country (Figure 1d). In addition to population aspects, it is important to emphasize that issues related to distribution logistics can influence the data used in the analysis.

**FIGURE 1:**
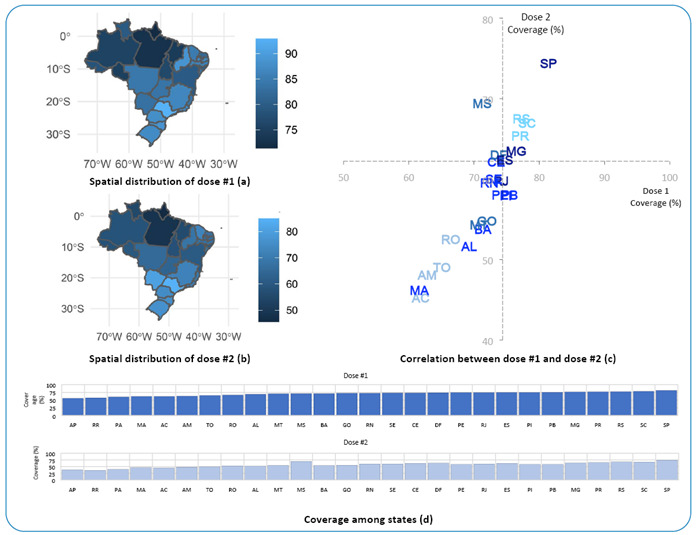
Descriptive analysis of vaccination coverage indicators by states according to dose in Brazil, 2021.

The vaccination strategy to mitigate the pandemic has been an effective measure, which translates into an increase in vaccination coverage over weeks. In this regard, it is essential to continue monitoring to detect any changes in the rising trend and understand the causes. There are two types of barriers to expanding immunization coverage. The first concerns systemic issues that affect a person's ability to access a service, including time, transport, cost, and clinic or point-of-sale location. On the other hand, behavioral barriers are related to attitudes towards vaccination and the extent to which beliefs or perceptions impact individuals’ willingness to adhere to vaccination^1^.

Even before the pandemic, there was growing evidence of delays or refusals of vaccines due to a lack of confidence in their importance, safety, and efficacy, particularly in Europe, which made room for the strengthening of anti-vaccine groups^5^. However, in general, the Brazilian population has been adhering to immunobiology. The excellent adherence to vaccination in Brazil may result from more than 40 years of building the credibility of the National Immunization Program, which guarantees universal coverage of the primary vaccination schedule in the country^6^. In this sense, it is reasonable to assume that stagnation in the country is more related to difficult access than the refusal to receive the vaccine.

Vaccination coverage is lower in poorer areas and ethnic minority groups. More recent analyses suggest that places with low development rates have lower coverage rates. We observed a 20% drop in the coverage of the first dose according to the level of development of the cities. Municipalities with a very high human development index (HDI) presented an immunization percentage of approximately 80% for the first dose. In contrast, in the group of cities with low HDI, this percentage was 60%. Some regions have an expressive part of their territory below the ideal vaccination rates. While the South and Southeast regions have a high percentage of the immunized population, areas in the North, Northeast, and Midwest regions still have pockets with low immunization for COVID-19. If we consider vaccination with a complete scheme above 80% as a safety scenario, we have only 16% of municipalities in Brazil in this situation^7^. Therefore, it is necessary to strengthen strategies committed to reducing the inequity of vaccination before new waves of infection become a priority.

It is worth noting that this indicator includes the total population in its denominator. In the reference period of the analysis, the population under 12 years of age was still not considered eligible for immunization in Brazil. However, immunizers with proven efficacy for this age group, and safety studies indicate that their use is possible^8^. There is an ongoing debate regarding whether all children under 12 years of age should be vaccinated against COVID-19. The relatively low risk posed by acute COVID-19 in children and uncertainty about the relative harm of vaccination and disease remains and fuels the debate's politicization.

In sheer numbers, children were least affected by COVID-19. However, this risk cannot be ignored. Brazil has insufficient availability of neonatal and pediatric intensive care unit (ICU) beds^9^; thus, even with a small contingent of sick children, the amount would be sufficient to potentially collapse pediatric and neonatal services, especially small and medium-sized cities. There are also population-level factors, such as reduced transmission from the community, the social cost of confinement of children, and other blocking measures. Thus, we can reach 85% of the population covered, considering only adolescents and adults. This is an optimal level, but it is far from ideal. Therefore, in addition to ensuring personal protection, vaccinating children is strategic for increasing vaccination coverage in Brazil.

Ultimately, it is essential to say that vaccination is an individual and collective responsibility^10^. Additionally, public confidence in the vaccine needs to be increased as its approval goes through many stages of technical validation. Thus, public trust is a significant barrier, and the debate surrounding the application of the COVID-19 vaccine in Brazil must be depoliticized. Furthermore, new strategies must be adopted to reach people in remote locations. At the same time, it is recommended to speed up the acquisition process of vaccines with proven safety among children aged 5-11 years, approved by the Brazilian Health Regulatory Agency (ANVISA) in December 2021; thus, this group is protected and, at the same time, allows for better total vaccination coverage. Finally, these actions are in line with a public policy commitment to reduce the inequity of vaccination and vaccinating children aged 5-11 years, expanding the country's total vaccination coverage.
